# Successful Clinical Outcome After a Modified Protocol of Rescue In Vitro Maturation of Oocytes: A Case Series Study

**DOI:** 10.7759/cureus.82927

**Published:** 2025-04-24

**Authors:** Nikos Petrogiannis, Maria Filippa, Kalliopi Chatzovoulou, Savvas Petrogiannis, Katerina Chatzimeletiou

**Affiliations:** 1 Assisted Reproductive Technology Unit, Naval Hospital of Athens, Athens, GRC; 2 Unit for Human Reproduction, 1st Department of Obstetrics and Gynaecology, Aristotle University of Thessaloniki, Thessaloniki, GRC

**Keywords:** blastocyst, clinical pregnancy, in vitro maturation, oocyte, rescue in vitro maturation

## Abstract

*In vitro* maturation (IVM) of oocytes represents an assisted reproductive technique with minimal or no ovarian stimulation application, rendering it beneficial for specific groups of patients facing infertility. IVM is based on the collection of immature cumulus-oocyte complexes (COCs) from antral follicles, subsequently cultured *in vitro* until they reach the metaphase II (MII) stage. Once maturation is accomplished, IVM oocytes are fertilized according to standard procedures. A variation of IVM is the “Rescue-IVM” protocol, which involves the maturation of immature COCs collected after a conventional *in vitro* fertilization (IVF) cycle, with a standard controlled ovarian stimulation protocol and *in vivo* human chorionic gonadotropin (hCG) maturation triggering. These immature COCs are cultured *in vitro* from the germinal vesicle (GV) on retrieval day (day zero) to the MII stage on the maturation check day (day one), prior to intracytoplasmic sperm injection (ICSI). In this study, we report five cases of patients who underwent an assisted reproductive technology (ART) cycle combined with modified Rescue-IVM, resulting in successful pregnancies. These cases offer high hope for patients with no synchronized follicles during ovarian stimulation to achieve a pregnancy, even when the fertilization occurs up to 28 hours post oocyte retrieval.

## Introduction

Since the birth of the first baby, Louise Joy Brown, conceived by *in vitro* fertilization (IVF), in 1978, many innovative events have taken place in the field of human assisted reproductive technologies (ARTs), attempting to improve clinical outcomes. A very promising alternative to conventional IVF has been introduced by reproductive scientists, through which immature cumulus-oocyte complexes (COCs) could mature *in vitro*, mimicking *in vivo* conditions, known as *in vitro* maturation (IVM) of oocytes. IVM involves the minimal or absence of ovarian stimulation, and it is beneficial to women with polycystic ovarian syndrome (PCOS) and/or patients who need a fertility preservation option before undergoing gonadotoxic treatment [1,2]. In fact, IVM was initially designed as an alternative to standard ovarian stimulation protocols to overcome the negative effects and risks associated with hormonal stimulation of ovaries, such as ovarian hyperstimulation syndrome (OHSS) in high responders. Advantages of IVM are the need for minimal medical monitoring, with fewer to no hormone injections, and lower costs. The basic IVM protocol is based on the collection of immature COCs from antral follicles that are subsequently cultured *in vitro* until they reach the metaphase II (MII) stage [3]. Once maturation in the laboratory is achieved, IVM oocytes are inseminated, fertilized, and treated exactly as the oocytes retrieved after a conventional IVF cycle [4]. Although IVM represents a hormone-free, easier, and less expensive procedure, both pregnancy rates and safety issues still remain questionable between IVF centers, making this subject a highly controversial one [5-8].

Concerning the IVM laboratory protocols, there are four major protocols that are currently practiced, some with *in vitro* human chorionic gonadotropin (hCG) and others with *in vivo* hCG triggering. The first one is the standard IVM protocol, which includes the collection of immature/germinal vesicle (GV)-stage COCs, which undergo IVM in a single step, until they reach the MII stage and are subsequently inseminated. The second one is the biphasic IVM protocol, which represents the evolution of the standard IVM. During the biphasic IVM, GV-stage COCs are cultured in a pre-IVM medium for 24 hours, where meiosis is inhibited at the GV stage, due to the presence of meiotic inhibitors in the culture medium. In the next step, oocytes finally mature from the GV to the MII stage, through meiosis-inducing factors [9]. The third one is the hCG-primed IVM or “Truncated” IVM protocol, during which patients are triggered *in vivo* with hCG to increase maturation outcome. In that way, it results in the presence of both immature and mature stage oocytes, which are inseminated at different time points in the laboratory [10]. The last one is the “Rescue-IVM'' or conventional IVF protocol, which includes the IVM of immature oocytes, both GV and metaphase I (MI) stage oocytes, collected after a conventional IVF cycle. These oocytes are commonly regarded as "incompetent" for clinical use and are usually discarded in such cycles, owing to their suboptimal quality. After oocyte retrieval and prior to insemination with intracytoplasmic sperm injection (ICSI), the oocytes are denuded of their cumulus cells, resulting in an invariable culture in a denuded state from the GV to MII stage in vitro. In fact, the aforementioned dubious oocyte quality puts into question the success of Rescue-IVM procedures [11], while in parallel, there is a widespread reporting of Rescue-IVM between clinics. Overall, both safety and effectiveness issues need to be addressed in future studies.

The present study aimed to evaluate the effectiveness and outcomes of a modified Rescue-IVM protocol designed to enhance *in vitro* oocyte maturation and embryo development in patients undergoing ART cycles.

## Case presentation

In our ART unit, we modified the Rescue-IVM protocol to achieve better maturation and capability of oocyte and embryo potentials. After the standard ovarian stimulation, *in vivo* hCG triggering, and COCs retrieval, the evaluation of maturity was done by the cumulus expansion appearance of the COCs. The COCs that were considered immature were left separately in culture, without being either denuded or inseminated, until the following day post retrieval, to evaluate their maturity status.

Here, we present five cases of patients who underwent an ART cycle combined with Rescue-IVM of oocytes in the Assisted Reproduction Unit of the Naval Hospital of Athens, Greece. This study is part of a PhD thesis that was approved by the scientific boards and ethics committees of the Naval Hospital of Athens, the Medical School of the Aristotle University of Thessaloniki, Greece, and the Greek National Authority of Assisted Reproduction.

Patient selection and informed consent

The patients included in this study had undergone at least one previous conventional ovarian stimulation (COS) IVF or ICSI treatment and fulfilled a number of criteria, while exclusion criteria have also been taken into account (Table 1). All patients signed an informed written consent for the Rescue-IVM protocol.

**Table 1:** Patient inclusion and exclusion criteria. AFC: antral follicle count; AMH: anti-Müllerian hormone; BMI: body mass index; FSH: follicle-stimulating hormone; WHO: World Health Organization.

Inclusion criteria	Exclusion criteria
Normal ovulatory women from 18 to 40 years old	Severe oligoteratospermia
Sperm parameters marginal to reference values of the WHO 2021 semen analysis manual	Patients with uterine disorders
Basal FSH values <10 mIU/mL, AMH values >1.1 ng/mL, AFC values >5-7 follicles	Natural cycle patients
BMI values between 18.5 and 30	Oocyte donated cycles or cryopreserved ova cycles

Clinical stimulation & laboratory fertilization protocol

All patients were treated by the gonadotropin-releasing hormone (GnRH) antagonist protocol, whereas recombinant follicle-stimulating hormone (r-FSH) was started on day two or three of the menstrual cycle (150-225 IU per day). When the leading follicle reached a size of at least 14 mm, a subcutaneous cetrotide acetate (Cetrotide, Merck Serono, Geneva, Switzerland) was administered at a dose of 0.25 mg/d. Then, when at least two to three follicles reached 17-18 mm mean diameter, a 10,000 IU rhCG (Ovidrel, Merck Serono, Geneva, Switzerland) was injected to trigger ovulation. At oocyte retrieval, 36 hours post hCG injection, COCs were collected by transvaginal ultrasound-guided follicle puncture. Husband's sperm sample was produced and processed on the same day, and IVF or ICSI was performed to mature oocytes, on day zero and/or day one, according to our modified Rescue-IVM protocol. The evaluation of the COCs' maturity status occurred four to five hours after oocyte pick-up (OPU), and if mature, they were either inseminated by IVF or denuded by using hyaluronidase supplemented with serum albumin (Hyase 10X, Vitrolife Group, Göteborg, Sweden), prior to ICSI. Immature COCs-oocytes were separated and cultured in standard culture medium, the Sequential Fert (Origio, Cooper Surgical, Ballerup, Denmark), in a standard CO2 6%, O2 5% incubator, until 20 to 28 hours post retrieval, by checking them whenever possible for their maturity status. When assessed mature, after Rescue-IVM, ICSI was followed on day one post retrieval, by recording everything in detail by date, time, COC number, status, and embryologist practice.

Embryo culture - vitrification - embryo transfer

**Figure 1:**
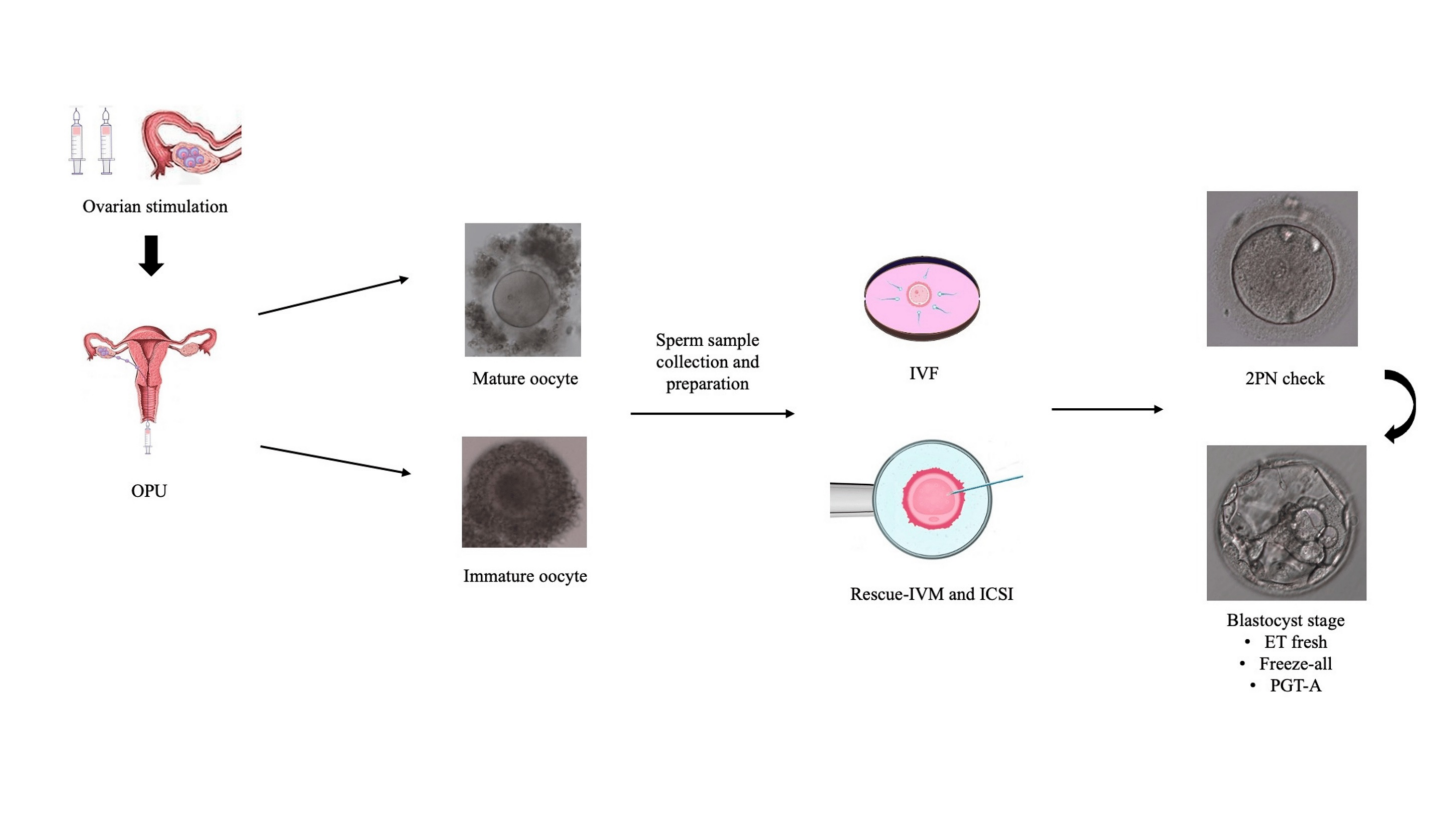
Laboratory workflow and modified Rescue-IVM protocol. ET: embryo transfer; ICSI: intracytoplasmic sperm injection; IVF: in vitro fertilization; IVM: in vitro maturation; OPU: oocyte pick-up; PGT-A: preimplantation genetic testing for aneuploidies; PN: pronuclei.

After insemination by IVF or ICSI on either day one or day two according to the modified Rescue-IVM, the oocytes were checked for fertilization at 16-18 hours post insemination, being considered as normally fertilized when two pronuclei (2PN) zygotes were visible. According to the ultrasound and hormonal profile of the patient, as well as the developmental rate, stage, and grade of the embryos, there was either a fresh day 5/6 embryo transfer or a freeze-all policy, accordingly. Embryos were evaluated by the corresponding stage of development, the fragmentation rate, and symmetry for cleavage-stage embryos, as well as their inner cell mass (ICM) and their trophoectoderm (TE) morphology for blastocyst-stage embryos. Grading scores of blastocysts were based on Gardner’s guidelines of embryonic morphological assessment [12]. Our modified Rescue-IVM protocol and laboratory workflow are summarized in Figure 1.

All embryos that were not transferred at the fresh cycle were vitrified using an open vitrification system (Kitazato, Fuji, Japan) and remained cryopreserved until a suitable endometrium was prepared. Vitrification of the embryos and "Freeze-all" strategy was indicated in patients at risk of OHSS, in cases when progesterone levels were elevated, as well as for medical and/or social reasons. Embryo thaw occurred according to Kitazato’s updated instructions. At the ART Unit of the Naval Hospital of Athens, frozen embryo transfer (FET) cycle endometrial preparation occurs with natural cycles (NC) and is performed three or five days after endometrial supplementation with luteal phase support until the 12th week of gestation. To evaluate clinical pregnancies, serum β-hCG tests were performed within 10-12 days after the embryo transfer. Live birth accounts for newborns with all four vital signs of respiration, heartbeat, umbilical cord pulsation, and random muscle contraction.

Five couples were referred to the ART Unit of the Naval Hospital of Athens for infertility treatments due to several factors, such as ovarian factor for three patients, repeated implantation failures (RIFs), and PCOS (Table 2). Patients underwent a COS protocol, combined with modified Rescue-IVM.

**Table 2:** Patient characteristics and full dataset. AFC: antral follicle count; AMH: anti-Müllerian hormone; BMI: body mass index; COS: controlled ovarian stimulation; ET: embryo transfer; FET: frozen embryo transfer; FSH: follicle-stimulating hormone; GnRH: gonadotropin-releasing hormone; hCG: human chorionic gonadotropin; ICSI: intracytoplasmic sperm injection; IVF: in vitro fertilization; IVM: in vitro maturation; LBR: live birth rate; NA: not applicable; PCOS: polycystic ovary syndrome; RIF: recurrent implantation failure.

	Patient 1	Patient 2	Patient 3	Patient 4	Patient 5
Patient characteristics					
Age (years)	39	34	33	40	28
Duration of infertility (years)	2	5	1.5	6	1
Cause of infertility	Ovarian	Ovarian	Ovarian	RIF	PCOS
BMI (kg/m^2^)	19.2	19	18.8	22.3	24.1
Basal FSH (mIU/mL)	4.7	5.2	6.1	4.8	4.6
AMH (ng/mL)	2	1.4	1.9	2.4	5.3
AFC	12	7	9	18	30
Sperm quality					
Total sperm concentration	35,000,000	22,000,000	40,000,000	16,000,000	32,200,000
Progressive motility (a+b)	34	36	38	35	40
Normal morphology (%)	4	4	5	4	5
COS characteristics					
COS protocol	Short antagonist	Short antagonist	Short antagonist	Short antagonist	Short antagonist
GnRH antagonist	Cetrorelix	Cetrorelix	Cetrorelix	Cetrorelix	Cetrorelix
COS duration (days)	10	11	10	12	11
COS dosage (IU)	225	225	225	225	150
Follicles >=14mm on hCG day (n)	13	6	7	16	28
Oocytes retrieved (n)	10	5	7	15	25
Before Rescue-IVM					
Mature oocytes (n)	2	2	2	4	8
Type of insemination (IVF/ICSI)	IVF	IVF	IVF	IVF	IVF
Fertilization rate (%)	0	50	50	50	50
Blastocyst rate (%)	0	100	0	100	50
Available embryos (n)	0	1	0	2	2
After Rescue-IVM					
Mature oocytes (n)	8	2	4	9	12
Type of insemination (IVF/ICSI)	ICSI	ICSI	ICSI	ICSI	ICSI
Fertilization rate (%)	75	100	75	66.6	66.6
Blastocyst rate (%)	66.6	100	66.6	50	75
Available embryos (n)	2	1	2	3	6
Fresh-ET	NA	NA	2	NA	NA
Freeze-all	2	1 + 1	NA	5	6
Fresh cycle					
Embryos ET (n)	NA	NA	2	NA	2
Good quality embryos (n)	NA	NA	2	NA	2
Implantation rate (%)	NA	NA	100	NA	0
Clinical pregnancy rate (%)	NA	NA	100	NA	0
LBR per ET (%)	NA	NA	100	NA	0
FET cycle					
Embryos ET (n)	2	1 + 1	NA	1	2
Good quality embryos (n)	2	2	NA	1	2
Positive β-hCG per ET (%)	100	100	NA	100	100
Clinical pregnancy rate (%)	100	100	NA	100	100
LBR per ET (%)	100	100	NA	100	100

Case 1

A 39-year-old woman with her husband was referred to our ART Unit due to ovarian factor infertility. The patient underwent a COS protocol, combined with modified Rescue-IVM. On the OPU day, 10 COCs were retrieved, and oocytes were split into two groups, namely, the “mature” (n = 2) and “immature” (n = 8) groups, according to the COCs' status. Mature oocytes were inseminated by IVF, while the immature oocytes were cultured overnight in standard culture conditions. The husband's sperm sample was processed on the same day prior to insemination (day zero). On day one, pronuclei (PN) check 18 hours post IVF revealed no signs of fertilization for none of the “mature” oocytes, as well as no further development was observed later on. The immature oocytes were first checked for their maturity status, and within 22 hours post retrieval, they were denuded, with the aid of hyaluronidase. Eight out of eight oocytes had reached the MII stage. ICSI was performed on all oocytes that had matured in vitro (n = 8). On day two, PN check revealed signs of normal fertilization with 2PNs appearance for six out of eight oocytes that had undergone Rescue-IVM. Zygotes cleaved normally, but only four had reached the blastocyst stage. Two blastocysts were of good quality (4BB and 3BB), according to Gardner’s guidelines of embryonic morphological assessment [12] and met quality criteria for vitrification, as was indicated by a freeze-all strategy for the patient. Blastocysts were vitrified and transferred in the patient’s next cycle, in a well-prepared endometrium, three hours post thawing. Ten days after the embryo transfer, a positive serum β-hCG test was obtained (150 IU), while five days later, the β-hCG was repeated (550 IU). This pregnancy resulted in a live birth of twins.

Case 2

A 34-year-old woman with her husband was referred to our ART Unit due to ovarian factor infertility. The patient underwent a COS protocol combined with Rescue-IVM. On the OPU day, five COCs were retrieved, and within two hours post oocyte retrieval, COCs were denuded to assess maturity status. From the five COCs retrieved, oocytes were split into two groups, namely, the “mature” (n = 2) and “immature” (n = 3) groups, according to the COCs' status. Mature oocytes were inseminated by IVF, while the immature oocytes were cultured overnight in standard culture conditions. The husband's sperm sample was processed on the same day prior to insemination (day zero). On day one, PN check 18 hours post IVF revealed normal signs of fertilization for one out of two “mature” oocytes. The immature oocytes were first checked for their maturity status, and within 22 hours post retrieval, they were denuded, with the aid of hyaluronidase. Two out of three oocytes had reached the MII stage, while the remaining one was arrested at the GV stage. ICSI was performed on all oocytes that had matured in vitro (n = 2). On day two, PN check revealed signs of normal fertilization with 2PNs appearance for both oocytes that had undergone Rescue-IVM. Zygotes cleaved normally, with three embryos reaching the blastocyst stage, one originating from the *in vivo* matured oocyte (blastocyst 1) and two from the *in vitro* matured oocytes (blastocysts 2 and 3). Two out of three blastocysts were of good quality (5AB for blastocyst No. 1, 4BB for blastocyst No. 2, and 2CC for blastocyst No. 3), according to Gardner’s guidelines of embryonic morphological assessment [12]. As a result, two blastocysts met quality criteria for vitrification, as was indicated by a freeze-all strategy for the patient. Both blastocysts were vitrified and transferred at different time points (two years between the two FETs), and both resulted in a live birth.

Case 3

A 33-year-old woman with her husband was referred to our ART Unit due to ovarian factor infertility. The patient underwent a COS protocol, combined with modified Rescue-IVM. On the OPU day, seven COCs were retrieved, with the majority of them being evaluated as immature, according to the COCs' status, four hours post OPU. Oocytes were therefore split into two groups, the “mature” (n = 2) and “immature” (n = 5) groups, with mature oocytes being inseminated by IVF, while the immature were cultured overnight in standard culture conditions. The husband's sperm sample was processed on the same day prior to insemination (day zero). On day one, PN check 18 hours post IVF revealed normal signs of fertilization for one out of two “mature” oocytes. The immature oocytes were first checked for their maturity status, and within 22 hours post retrieval, they were denuded, with the aid of hyaluronidase. Four out of five oocytes had reached the MII stage, while the remaining one was at the MI stage. ICSI was performed on all oocytes that had matured in vitro (n = 4). On day two, PN check revealed signs of normal fertilization with 2PNs appearance for three out of four oocytes that had undergone Rescue-IVM. Zygotes cleaved normally, although the embryo originating from the *in vivo* matured oocyte did not reach the blastocyst stage (checked on days five and six). Two embryos originating from the *in vitro* matured oocytes had reached the blastocyst stage, which were both morphologically ideal for transfer (6AA and 4AB) and were therefore transferred in a fresh cycle on day five. A positive serum β-hCG test was obtained 12 days after the embryo transfer (ET, 500 IU), while six days later, the β-hCG was repeated and was found to be increased (900 IU). This pregnancy resulted in a live birth of twins.

Case 4

A 40-year-old woman with her husband was referred to our ART Unit due to a RIF infertility case. The patient had a history of three implantation failures, two resulting from natural conception and one after an IVF cycle. The couple was therefore referred to our center to proceed with a COS protocol, combined with preimplantation genetic testing for aneuploidies (PGT-A) to assess the ploidy status of the embryos, before embryo transfer. A written consent was given by the Greek National Authority of Assisted Reproduction to perform PGT-A on the embryos. On the OPU day, 15 COCs were retrieved, with the majority of them being evaluated as immature, according to the COCs' status, four hours post OPU. Oocytes were therefore split into two groups, the “mature” (n = 4) and “immature” (n = 11) groups, with mature oocytes being inseminated by IVF, while the immature were cultured overnight in standard culture conditions. The husband's sperm sample was processed on the same day prior to insemination (day zero). On day one, PN check 18 hours post IVF revealed normal signs of fertilization for only two out of four mature oocytes. The immature oocytes were first checked for their maturity status, and within 22 hours post retrieval, they were denuded, with the aid of hyaluronidase. Nine out of 11 oocytes had reached the MII stage, while the remaining two were at the MI stage. ICSI was performed on all oocytes that had matured *in vitro* (n = 9). On day two, PN check revealed signs of normal fertilization with 2PNs appearance for six out of nine oocytes that have undergone Rescue-IVM. Zygotes cleaved normally, with five embryos reaching the blastocyst stage, two originating from the *in vivo* matured oocytes (blastocysts 1 and 2) and three from the *in vitro* matured oocytes (blastocysts 3, 4, and 5). All blastocysts were biopsied on day five, and trophectoderm samples were tubed and sent to the genetic laboratory for aneuploidy assessment by next-generation sequencing (NGS). Each embryo was vitrified separately on a single carrier/straw (Kitazato, Fuji, Japan) to identify the euploid embryos, after the PGT-A result, and proceed with the FET cycle. According to the NGS analysis, blastocysts 1, 2, and 4 presented multiple aneuploidies, while blastocysts 3 and 5 were found to be euploid and therefore suitable for transfer. As a result, blastocyst 3 was transferred in the patient’s next cycle, in a well-prepared endometrium, three hours post thawing. Ten days after the embryo transfer, a positive serum β-hCG test was obtained (350 IU). As indicated, amniocentesis was performed on the 15th week of pregnancy, and no genetic abnormality was detected. This pregnancy resulted in a live birth. The patient has one remaining day five euploid blastocyst (blastocyst No. 5) cryopreserved in the cryopreservation bank of our clinic.

Case 5

A 28-year-old woman with her husband was referred to our ART Unit due to the PCOS factor of infertility. The patient underwent an ovarian stimulation protocol with a low dose of gonadotrophins (150 IU), combined with modified Rescue-IVM, due to the expected increased number of immature oocytes. On the OPU day, 25 COCs were retrieved, with the majority of them being evaluated as immature, according to the COCs' status, four hours post OPU. Oocytes were therefore split into two groups, the “mature” (n = 8) and “immature” (n = 17) groups, with mature oocytes being inseminated by IVF, while the immature were cultured overnight in standard culture conditions. The husband's sperm sample was processed the same day prior to insemination (day zero). On day one, PN check 18 hours post IVF revealed normal signs of fertilization for four out of eight “mature” oocytes. The immature oocytes were first checked for their maturity status, and within 21 hours post retrieval, they were denuded, with the aid of hyaluronidase. Twelve out of 17 oocytes had reached the MII stage, while the remaining five were at the MI stage. ICSI was performed on all oocytes that had matured *in vitro* (n = 12). On day two, PN check revealed signs of normal fertilization with 2PNs appearance for eight out of 12 oocytes that have undergone Rescue-IVM. Zygotes cleaved normally, and embryos were observed at day three for morphological assessment until they reached the blastocyst stage. From the “mature” group of oocytes, only two embryos had reached the blastocyst stage and were suitable for transfer (ET fresh, 4AB and 5BC blastocysts). A negative serum β-hCG test was obtained 12 days after the ET. From the “immature” group of oocytes that had matured *in vitro*, six embryos reached the blastocyst stage and met quality criteria for vitrification. Blastocysts were vitrified in pairs, and the first set of embryos was transferred in the patient’s next cycle, in a well-prepared endometrium, three hours post thawing. Ten days after the embryo transfer, a positive serum β-hCG test was obtained (850 IU), while five days later, the β-hCG was repeated (1150 IU). This pregnancy resulted in a live birth of twins. The patient has four remaining day five embryos cryopreserved in the cryopreservation bank of our center.

## Discussion

*In vitro* maturation of oocytes is supposed to be an advantageous and promising technique, especially for patients with specific indications, as it requires minimal or absence of ovarian stimulation, less time, as well as minimal medical monitoring [13]. A variation to the IVM approach, the so-called “Rescue-IVM” protocol, involves the culture of immature oocytes collected from conventional IVF cycles, which are typically discarded, due to their state (GV and/or MI oocytes). During the “Rescue-IVM” protocol, oocytes are commonly denuded of cumulus cells at oocyte pick-up to check maturity, so immature oocytes are typically matured from GV to MII in the absence of cumulus cell support. Such oocytes are of dubious quality, thus making the procedure questionable [11]. In fact, the controversies and variability in the outcomes of Rescue-IVM procedures that have been observed might be explained by the uncertain origin of the GV and/or MI oocytes, creating limitations in the use of IVM. For instance, when oocytes mature outside of the follicular environment, serious complications might be introduced. The doubtful origin of such oocytes might be the result of an abnormal or failed reaction to the ovulatory trigger, the oocyte’s extraction from small and therefore incompetent follicles, as well as an early atretic one [14]. As a result, such findings have restrained most clinics from the systematic use of these oocytes, as chromosomal analysis of the immature oocytes after standard ovarian stimulation indicated aneuploidies, or risk of spindle misalignment, cytoplasmic and genetic abnormalities, as well as potential epigenetic changes [15,16], adding a general skepticism on its clinical use. On the other hand, some scientific groups have reported promising results after the application of both IVM [17] and Rescue-IVM procedures. More specifically, after the application of Rescue-IVM, scientists were able to obtain blastocysts for research purposes [18], as well as pregnancies and live births [19]. In fact, in a retrospective study, based on 2602 patients, the authors reported that adding Rescue-IVM technology to regular cycles resulted in increased pregnancy rates by 6% overall [19]. Interestingly, a more recent randomized controlled trial (RCT) by Vuong and colleagues demonstrated that the application of hormone-free infertility treatment by using a biphasic IVM protocol was beneficial in women with PCOS, especially in terms of ongoing pregnancies and live birth rates [20].

Here, we report five cases that were referred to our center for several infertility factors, such as ovarian factor, RIF, and PCOS. All patients underwent a conventional ovarian stimulation protocol, combined with modified (when applicable) Rescue-IVM, due to an expected low number of mature oocytes at OPU and according to the patient’s history of infertility. In all cases, the use of Rescue-IVM was beneficial and even more efficient than the conventional IVF protocol in the patient’s same cycle. In fact, we were able to collect immature oocytes, culture them in standard conditions, and thus achieve maturation at a high rate, compared to the conventional IVF cycle yield. All embryos originating from the “immature” group of oocytes resulted in a clinical pregnancy and live birth.

## Conclusions

Overall, our work emphasizes the positive outcome of the use of Rescue-IVM technology, especially in patients with a low number of mature oocytes at oocyte retrieval. In the future, well-designed clinical trials, larger prospective trials (case-control studies), and retrospective cohort studies to compare conventional IVF and modified Rescue-IVM protocols would be of great importance. These studies will help contextualize the effectiveness of the modified protocol and provide a cost-effectiveness analysis. Finally, they would provide more data on the beneficial results of the use of Rescue-IVM in clinical practice, aiming to achieve increased clinical pregnancy and increased live birth rates.
